# Case Report: Acute cerebral infarction caused by left atrial and right ventricular myxoma received emergency operation

**DOI:** 10.3389/fcvm.2023.1316063

**Published:** 2024-01-12

**Authors:** Chengbin Tang, Xianglong Gao, Tao Chen, Jun Shao, Tao Zhu, Xucai Zheng, Chuanli Ren

**Affiliations:** ^1^ Department of Cardiovascular Surgery, Northern Jiangsu People's Hospital Affiliated to Yangzhou University, Yangzhou, China; ^2^ The Yangzhou Clinical Medical College of Xuzhou Medical University, Xuzhou, China; ^3^ Department of Breast and Thyroid Surgery, The First Affiliated Hospital of University of Science and Technology of China, Hefei, China; ^4^ Department of Laboratory Medicine, Clinical College of Yangzhou University, Yangzhou, Jiangsu, China

**Keywords:** myxomas, neurologic symptoms, emergency operation, case report, cardiac tumors

## Abstract

Cardiac myxoma is a rare etiology of ischemic stroke, especially in young people. We report a case of multiple myxomas in left atrium and right ventricle inducing acute cerebral infarction. No significant abnormalities were detected in the patient's preoperative laboratory examination. Following emergency surgery, the patient's prognosis was satisfactory, providing valuable empirical insight for the surgical management of acute cerebral infarction in individuals diagnosed with cardiac myxoma. Our objective is to heighten awareness regarding the evaluation and treatment of patients with acute cerebral infarction subsequent to early diagnosis of cardiac myxoma.

## Introduction

Cardiac myxomas account for between 50% and 85% of all cardiac tumors (1). Sixty percent to 80% of cardiac myxomas occur in the left atrium, usually attached to fossa ovalis, the right atrium and ventricle and valves are relatively rare (1, 2). Controversy surrounds the treatment of neurologic symptoms caused by cardiac myxoma, focusing on whether to prioritize removing cardiac tumors or addressing cerebral infarction first.

**Figure 1:**
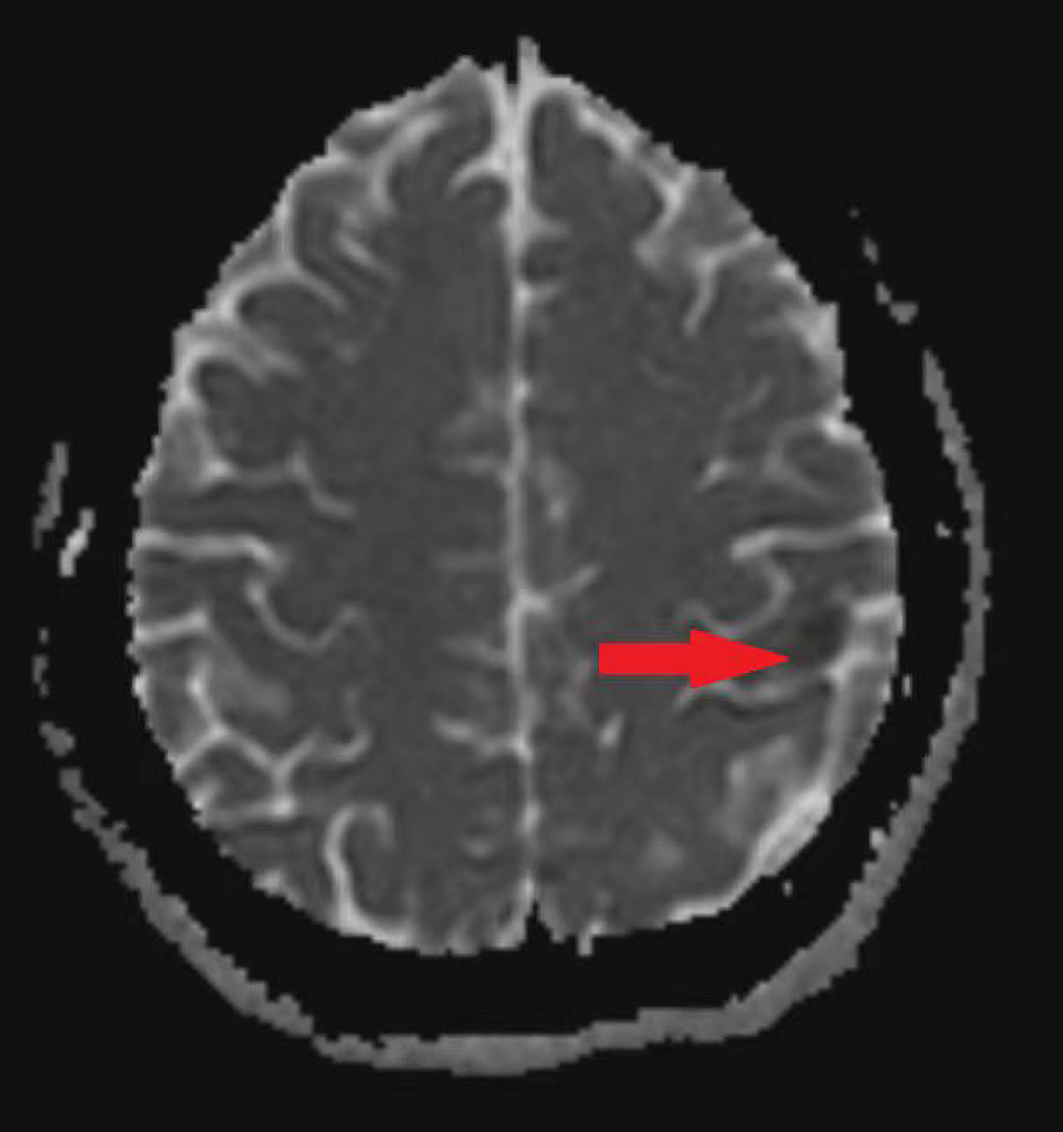
The MRI of the patient's head before operation, the arrow is the focus area.

**Figure 2:**
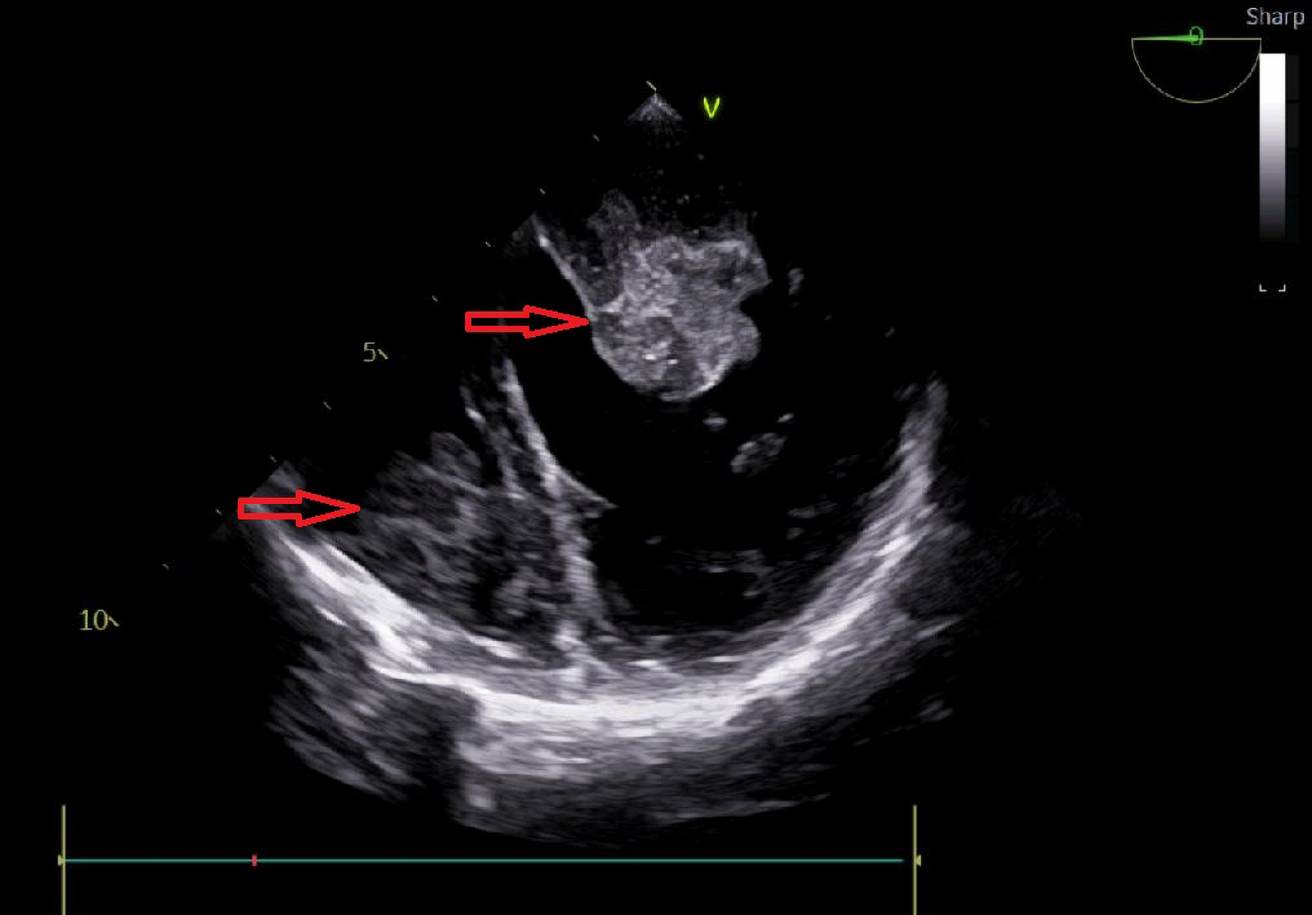
Transesophageal echocardiography, the mass shadow at the arrow is myxoma.

**Figure 3:**
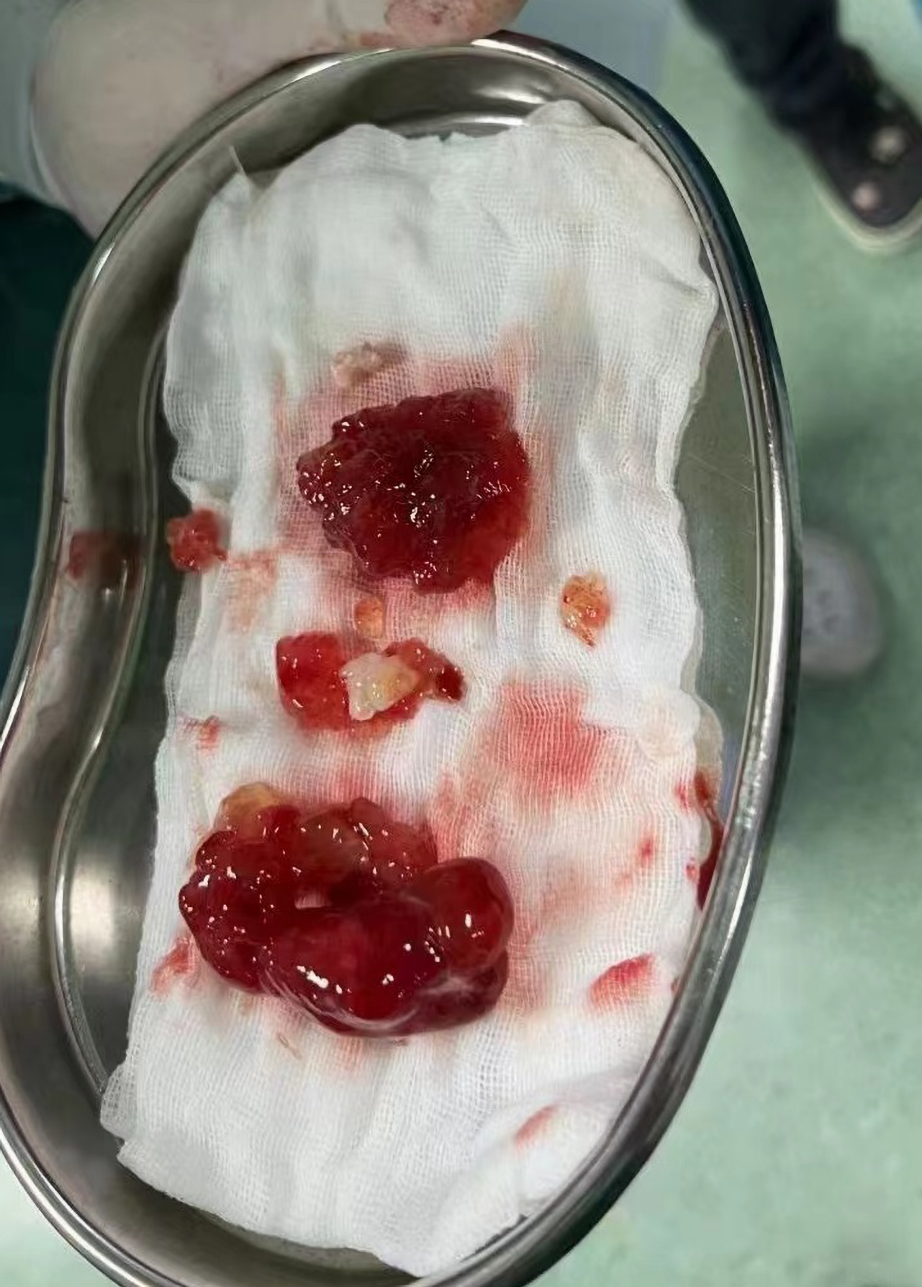
The red jelly-like substance in the bowl is the tissue of myxoma removed by surgery.

**Figure 4:**
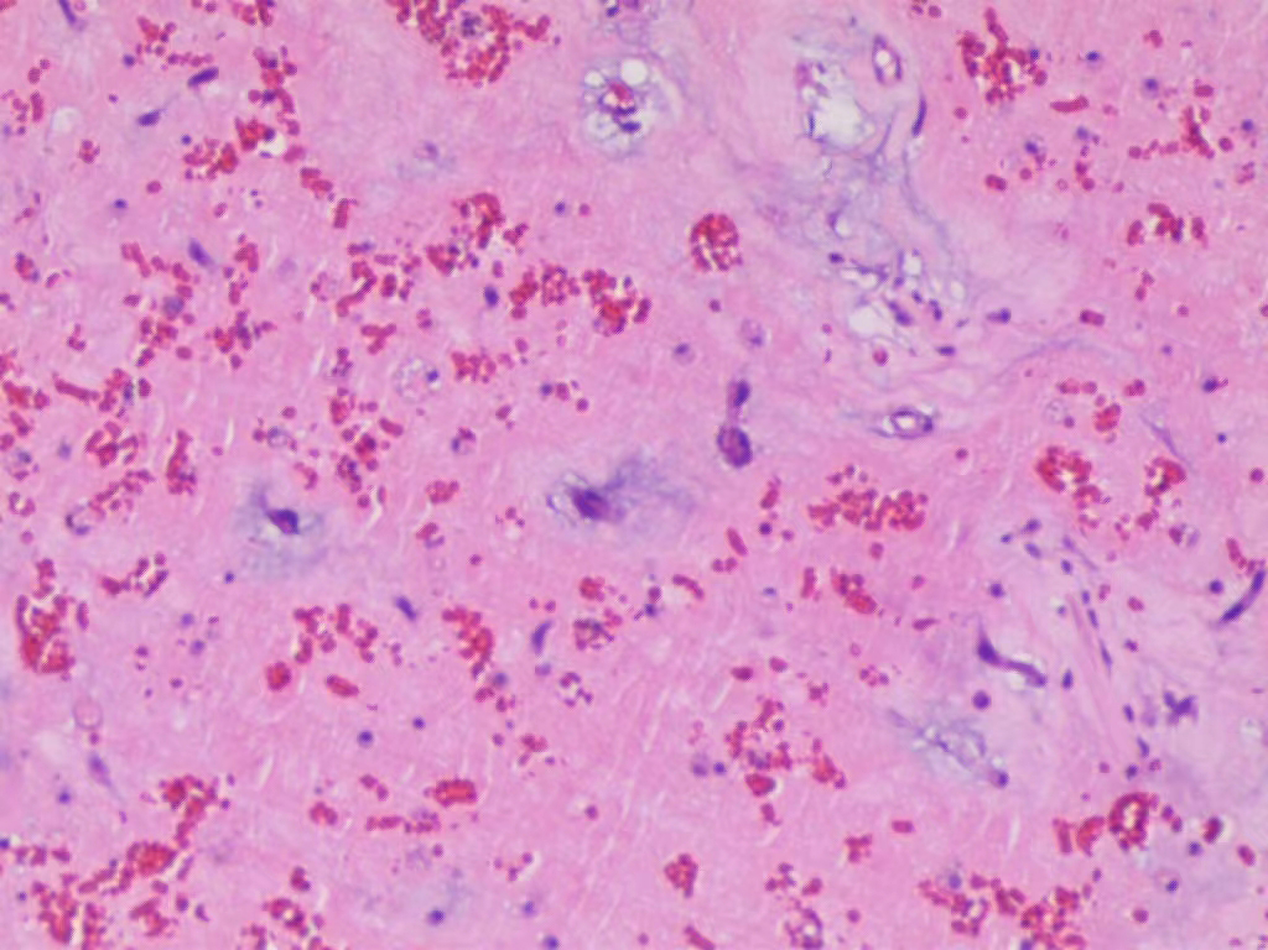
Sections under microscopic examination showed that the tumor cells are scattered in the myxoid matrix, star-shaped and polygonal, diffuse or intertwined into a network, and the focal area bleeds.

A 25-year-old woman came to our emergency center with repeated chest tightness, palpitations, asthma, occasional hand and foot numbness, and worsening symptoms during daily activities over a 2-month period. Dizziness, headache, nausea and vomiting occurred 1 day before hospital admission. A magnetic resonance imaging (MRI) of the head revealed multiple specks and flaky signals in the left cerebral hemisphere, suggesting the possibility of multiple acute cerebral infarctions (Figure 1, arrow). Intraoperative transesophageal ultrasound demonstrated a soft mass shadow attached to the anterior lobe of the mitral valve, which collided with the left ventricle during diastole. Additionally, another mass shadow was observed at the apex of the right ventricle with minimal activity (Figure 2, arrow below). Subsequently, a dark red jelly-like mass, sized approximately 5 × 3 cm in the left atrium and 3 × 2 cm in the right ventricle, was identified and completely excised (Figure 3). The histopathological examination of a biopsy specimen confirmed the presence of cardiac myxomas (Figure 4).

Ten hours after admission, the patient underwent an emergency operation in which a median sternal incision was made to gain access to the chest. Heparinization of the entire body was then carried out, followed by the establishment of cardiopulmonary bypass through the ascending aorta and superior and inferior vena cava. Subsequently, the entire body was cooled, and the superior and inferior vena cava were blocked. The left atrium was then incised and drained, with temporary omission of the left atrial tube insertion, to prevent the myxoma from dislodging. The ascending aorta was then blocked and cardioplegia was infused through the aortic root before a longitudinal incision was made in the right atrium and atrial septum following cardiac arrest. During the procedure, a dark red jelly-like mass, measuring approximately 5 × 3 cm, was identified in the left atrium and successfully removed. A left atrial retractor was then used to open the anterior lobe of the tricuspid valve, revealing a dark red jelly-like tumor measuring about 3 × 2 cm in the right ventricle. Following its removal, the tumor was meticulously rinsed multiple times with warm water to ensure no residual tumor was left in any cardiac cavity, thereby minimizing the risk of recurrence (3). The procedure lasted a total of 76 min under cardiopulmonary bypass, with 49 min being cardiac function-blocked. Post-operation, the patient was transferred to the intensive care unit with endotracheal intubation, and regained consciousness 3 h later. The endotracheal tube was successfully removed on the second day after the operation.

The patient was discharged from the hospital 10 days after surgery in good spirits and the muscle strength of the limbs was normal. Despite the successful recovery in physical strength, there was an observed temporary decrease in the patient's calculation ability. However, during the 3-month follow-up, the patient exhibited no residual dysarthria or difficulty accessing words, and there were no recurrent ischemic events. Additionally, her calculation ability had returned to normal.

## Discussion

As per our literature search, this is the first case report of multiple myxomas in left atrial and right ventricular inducing acute cerebral infarction in a young female patient. In this case, the patient was young and had cerebral infarction symptoms, which reminds doctors to pay attention to cardiogenic factor (4). In addition, the preoperative head magnetic resonance (MRI) of the patient showed that the areas of cerebral infarction were not confined to the same vascular area, which suggested that the stroke originated from the heart (5).

Cardiac MRI can provide unique information in the diagnosis of cardiac myxoma with robust tissue signature sequences (6). In this case, the patient suffered from acute cerebral infarction caused by cardiac myxomas. In order to prevent further aggravation of obstruction induced by cardiac myxomas, we performed emergency surgery as soon as possible to remove the risk of re-obstruction. The treatment of neurologic symptoms resulting from cardiac myxoma-induced infarction is a subject of debate, with no established guideline for stroke associated with myxoma (7). However, some scholars argue that immediate treatment of cardiac myxoma upon diagnosis is crucial for averting re-vascular obstruction, prioritizing it over the alleviation of neurological or other embolic symptoms in the brain (8).

Some scholars support the priority use of intravenous thrombolytic drugs after cerebral embolism, with recombinant tissue plasminogen activator considered safe and effective. However, postoperative pathology findings in successful thrombolysis cases revealed that the main component of the embolus was thrombosis. This finding may be attributed to the local vascular ischemic damage caused by myxoma embolus blocking cerebral blood vessels (9).

The choice of operation time window has also become a controversial topic. Wakako Fukuda et al. (10) conducted a study revealing that the occurrence of worsening brain complications decreased by 10% when surgery was performed more than 15 days after cerebral infarction. In the case reported by Yoshioka et al. (11), the patient's preoperative cranial MRI showed only multiple small infarcts in the brain without any signs of cerebral hemorrhage. After emergency surgery, right occipital lobe cerebral hemorrhage was found, notably, the patient's preoperative test results, including a platelet count of only 1 × 10^4^/mm^3^ and a D-dimer level of 12.72 μg/ml, indicated severe disseminated intravascular coagulation (DIC). This case emphasizes the critical nature of preoperative coagulation function examination to avoid unforeseen complications during surgery.

In this case, after surgically removing the risk of cardiogenic obstruction, the neurological symptoms improved, and there were no new obstruction symptoms in other organs. Therefore, we believe it is crucial to promptly remove cardiac myxoma in non-DIC patients.

## Data Availability

The original contributions presented in the study are included in the article/Supplementary Material, further inquiries can be directed to the corresponding author.
